# Linear array detector for online diagnostics of spectral distributions at MHz repetition rates^1^

**DOI:** 10.1107/S1600577519007835

**Published:** 2019-09-01

**Authors:** Christopher Gerth, Günter Brenner, Michele Caselle, Stefan Düsterer, Daniel Haack, Dariusz Makowski, Aleksander Mielczarek, Steffen Palutke, Lorenzo Rota, Vladimir Rybnikov, Christian Schmidt, Bernd Steffen, Kai Tiedtke

**Affiliations:** a Deutsches Elektronen-Synchrotron (DESY), Hamburg, Germany; b Karlsruhe Institute of Technology (KIT), Eggenstein-Leopoldshafen, Germany; c Lodz University of Technology, Department of Microelectronics and Computer Science, Lodz, Poland

**Keywords:** linear array detector, line scan camera, high repetition rate diagnostics, free-electron laser, extreme ultraviolet radiation, soft X-ray spectroscopy

## Abstract

Free-electron lasers (FELs) based on superconducting accelerator technology and storage ring facilities operate with bunch repetition rates in the MHz range, and the need arises for bunch-by-bunch electron and photon diagnostics. For photon-pulse-resolved measurements of spectral distributions, fast one-dimensional profile monitors are required. The linear array detector KALYPSO (KArlsruhe Linear arraY detector for MHz-rePetition rate SpectrOscopy) has been developed for electron bunch or photon pulse synchronous read-out with frame rates of up to 2.7 MHz. At the FLASH facility at DESY, a current version of KALYPSO with 256 pixels has been installed at a grating spectrometer as online diagnostics to monitor the pulse-resolved spectra of the high-repetition-rate FEL pulses. Application-specific front-end electronics based on MicroTCA standard have been developed for data acquisition and processing. Continuous data read-out with low latency in the microsecond range enables the integration into fast feedback applications. In this paper, pulse-resolved FEL spectra recorded at 1.0 MHz repetition rate for various operation conditions at FLASH are presented, and the first application of an adaptive feedback for accelerator control based on photon beam diagnostics is demonstrated.

## Abstract 2

The novel line detector KALYPSO has been developed for the measurement of one-dimensional profiles at high-repetition-rate free-electron lasers (FELs) and synchrotron radiation facilities. The current version has 256 pixels with a continuous data readout at a maximum frame rate of 2.7 MHz. At FLASH, KALYPSO has been utilized as photon diagnostics for monitoring pulse-resolved FEL spectra at a repetition rate of 1.0 MHz. KALYPSO is a collaborative effort between the Karlsruhe Institute of Technology (KIT), Paul Scherrer Institut (PSI), Lodz University of Technology (TUL-DMCS) and Deutsches Elektronen-Synchrotron (DESY).

## 1. Introduction   

At high-repetition-rate free-electron lasers (FELs) (Wiedorn *et al.*, 2018 ▸; Faatz *et al.*, 2016 ▸) and ultra-low-emittance storage rings (Tavares *et al.*, 2018 ▸), beam diagnostics with single-bunch or bunch-by-bunch resolution become increasingly important for stable operation especially in applications of fast feedback control loops. Photon diagnostics is of equal importance when variations of photon pulse properties have an impact on the measurement results of user experiments. This is particularly the case for single-pass FELs generating FEL radiation based on self-amplified spontaneous emission (SASE). Since the exponential amplification process in a SASE FEL starts from spontaneous emission in the electron bunch (shot noise), the SASE FEL radiation is inherently of stochastic nature, *i.e.* individual radiation pulses differ in intensity, spectral distribution and temporal structure (Ayvazyan *et al.*, 2003 ▸; Düsterer *et al.*, 2014 ▸). In addition, drifts or instabilities of electron beam parameters, *e.g.* beam energy, beam emittance, bunch charge, *etc*., lead to variations of the photon pulse properties. Hence, electron beam and photon pulse diagnostic tools that operate in a non-destructive way with single-shot resolution are of fundamental importance for both the optimization and control of the FEL radiation as well as evaluation and interpretation of measurement results obtained in user experiments.

**Figure 1:**
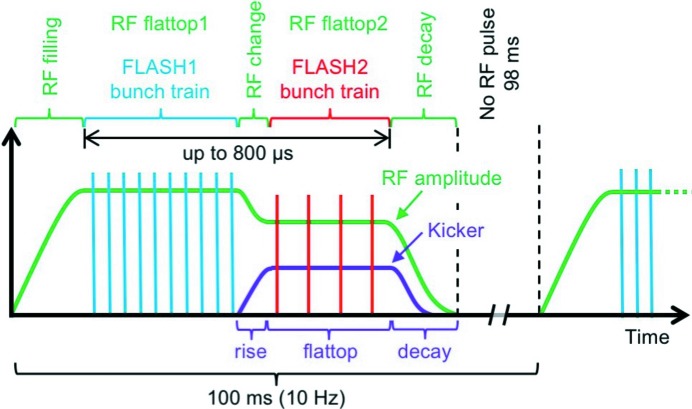
Simplified diagram of the FLASH timing pattern. The RF accelerating pulses (green line) have a repetition rate of 10 Hz and are split into two flattop regions for the FLASH1 (blue bars) and FLASH2 (red bars) bunch trains. The number of bunches and their repetition rate, at maximum 1.0 MHz, can be adjusted independently. A kicker-septum system (purple line) is used to deflect the FLASH2 bunch train into the FLASH2 FEL beamline.

At the FLASH facility (Ackermann *et al.*, 2007 ▸), both FEL beamlines FLASH1 and FLASH2 are driven by one superconducting linear accelerator that operates in bunch-train mode with electron beam energies of up to 1.25 GeV (Faatz *et al.*, 2016 ▸). An exemplary electron bunch pattern, or respective photon pulse pattern, is depicted schematically in Fig. 1 ▸. The radio-frequency (RF) accelerating pulses (solid green line), which have a length of up to 800 µs and a repetition rate of 10 Hz, can be split into two flattop regions for parallel operation of the FLASH1 and FLASH2 beamlines with 10 Hz. Both flattop regions can be filled independently with a freely selectable number of bunches at a maximum repetition rate of 1.0 MHz (blue and red bars). The gap of about 30–50 µs between the flattop regions is used for adjustment of the RF accelerating voltage and phase, depending on the operation mode of FLASH2, and for the rise-time of a fast kicker-septum system (purple line) that deflects the bunches within the second flattop region into the FLASH2 beamline.

FLASH1 and FLASH2 are single-pass SASE FELs generating soft X-ray and extreme ultraviolet (XUV) laser radiation in the wavelength ranges 4.2–52 nm and 4.0–90 nm, respectively (Faatz *et al.*, 2016 ▸). Monitoring of the photon pulse energies in absolute terms is achieved with gas-monitor detectors (GMDs) (Tiedtke *et al.*, 2008 ▸; Richter *et al.*, 2003 ▸), which are indispensable for FEL tuning as well as user experiments that study non-linear effects in light–matter interactions. User experiments that are highly sensitive to the wavelength of the FEL radiation, *e.g.* at ionization edges or resonances, demand detailed knowledge of the spectral distributions of the individual FEL pulses. However, fast line scan cameras with MHz frame rates and continuous data read-out are not available commercially. The recording of up to about 100 FEL spectra at a repetition rate of 800 kHz was achieved at FLASH (Palutke *et al.*, 2015 ▸) with the GOTTHARD detector (Mozzanica *et al.*, 2012 ▸), which stores the spectra in a local memory prior to read-out between pulse trains. The resulting read-out latency and limited number of frames render continuous data recording and applications for fast feedbacks impossible.

In this paper, we present first results obtained with the linear array detector KALYPSO (Rota *et al.*, 2019 ▸) utilized at the variable-line-spacing grating spectrometer (Brenner *et al.*, 2011 ▸) in the FLASH1 beamline for monitoring the spectral distributions of FEL radiation pulses at MHz repetition rates. KALYPSO (KArlsruhe Linear arraY detector for MHz-rePetition rate SpectrOscopy) is a line scan detector with 256 pixels for visible and near-infrared radiation that operates at a continuous frame rate of up to 2.7 MHz in streaming mode with low latency in the microsecond range.

## 2. Linear array detector system   

**Figure 2:**
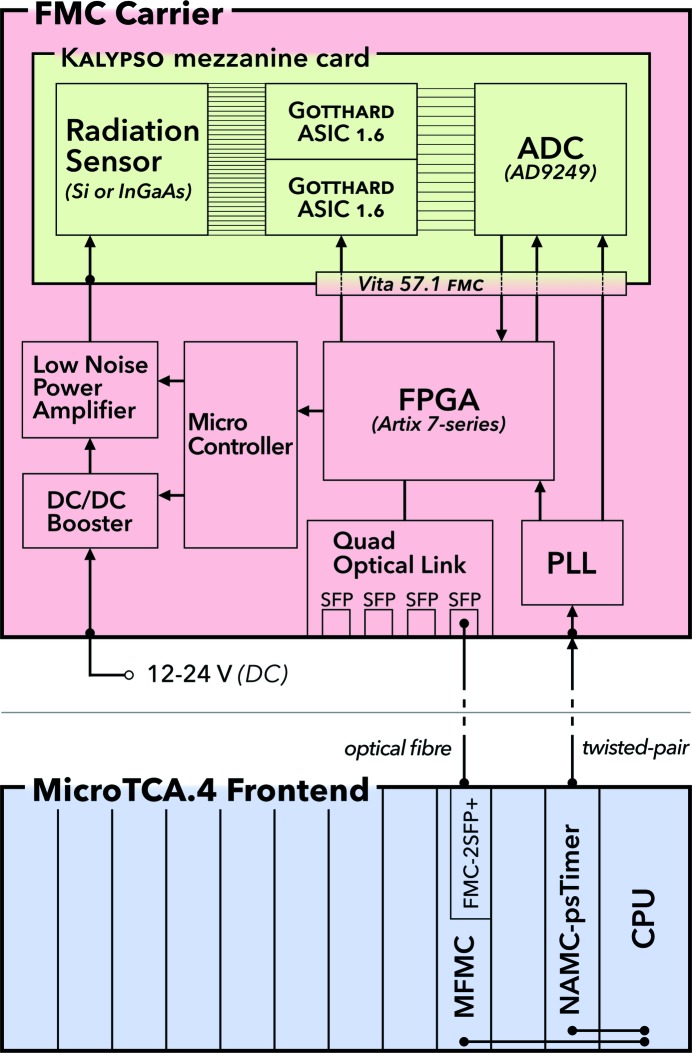
Block diagram of the linear array detector system. The KALYPSO mezzanine card is hosted on the FMC carrier for data acquisition and control. The FMC carrier is connected to the FLASH front-end electronics in MicroTCA.4 standard via optical fibres (data and control) and twisted-pair cables (timing signals) which can have a length of up to a few hundred meters.

The detector system comprises a modular architecture that consists of three main components: (i) the KALYPSO mezzanine card with the radiation sensor and analog-to-digital signal conversion, (ii) the carrier for the KALYPSO mezzanine card equipped with a field programmable gate array (FPGA) for data acquisition and processing, and (iii) the accelerator front-end electronics in Micro Telecommunication Computing Architecture (MicroTCA) standard for integration into the control system and synchronization to the FLASH timing system. A block diagram of the detector system, which is described in more detail in the following subsections, is depicted schematically in Fig. 2 ▸. This modular approach has the advantage that application-specific KALYPSO mezzanine cards can be combined with custom-made FPGA carrier boards for data acquisition and integration into specific control systems at different facilities. It also separates the radiation detection part from the data acquisition part enabling almost independent further development of both components. The signal transmission via optical fibres and twisted-pair cables permits the installation of the FPGA carrier with the KALYPSO mezzanine card up to a few hundred meters away from the accelerator front-end electronics.

### 2.1. KALYPSO mezzanine card   

The KALYPSO mezzanine card (Rota *et al.*, 2019 ▸), which is depicted as a green area in the block diagram in Fig. 2 ▸, is a printed circuit board (PCB) with a size of 120 mm × 90 mm that comprises the radiation sensor, analog signal processing and analog-to-digital converters (ADCs).

For the detection of X-rays as well as for radiation in the visible and up to wavelengths of about 1050 nm, an Si microstrip sensor is used. The sensor has a total of 256 microstrips with a channel pitch of 50 µm and a height of 8 mm. Alternatively, a commercial InGaAs microstrip sensor (Xenics), which is sensitive in the 0.9–1.7 µm wavelength range, can be mounted.

The microstrips of the Si sensor are connected via Au ball-to-wedge wire bonding to the input channels of two front-end ASICs (Application Specific Integrated Circuits) which are modified versions of the GOTTHARD chip (Mozzanica *et al.*, 2012 ▸). Each GOTTHARD ASIC comprises 128 input channels, each of which consists of a charge-sensitive amplifier (CSA) and a channel buffer. An analog 16:1 multiplexer combines 16 input channels to one differential output driver resulting in eight output channels per GOTTHARD ASIC. The 16 analog outputs of both GOTTHARD ASICs are connected to the PCB via standard Al wedge-to-wedge wire bonding and routed to a commercial 16-channel ADC (AD9249, Analog Devices) with 14-bit resolution that is operated at a sampling rate of 54 MHz. Each ADC output channel is routed to a VITA 57.1 FMC connector (VITA, 2010 ▸) which enables the KALYPSO mezzanine card to be placed on a FPGA mezzanine card (FMC) carrier board. In order to achieve a higher analog bandwidth with respect to the original GOTTHARD chip, the correlated-double-sampling (CDS) stage and automatic gain switching mechanism have been omitted. In this way, a maximum frame rate of 2.7 MHz has been achieved (Rota *et al.*, 2016 ▸), which is an improvement of almost a factor of three with respect to the original GOTTHARD design. As a consequence, the higher bandwidth and lack of CDS noise shaper result in a higher noise contribution.

The KALYPSO mezzanine card is also equipped with digital-to-analog converters (DACs) to provide bias voltages for the GOTTHARD ASICs and a clock conditioner for the generation of a local low-jitter clock synchronous to an input clock. However, in the configuration described in this paper, the clock and timing signals for the ADC and GOTTHARD ASICs as well as the bias voltage for the radiation sensor were generated on the FMC carrier board and supplied to the KALYPSO mezzanine card.

### 2.2. FMC carrier for data acquisition and processing   

**Figure 3:**
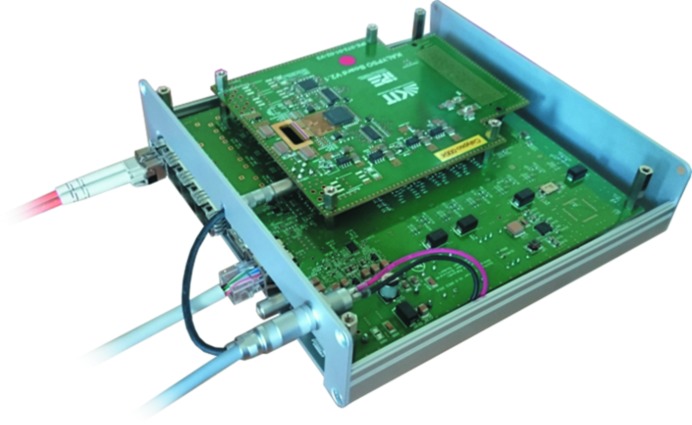
Photograph of the FMC carrier equipped with the KALYPSO mezzanine card.

The KALYPSO mezzanine card is connected via the VITA 57.1 FMC connector to an FMC carrier. This FMC carrier (red area in Fig. 2 ▸) incorporates a FPGA (Xilinx, 7-series Artix) and a Double Data Rate 3 (DDR3) memory which are used for the data acquisition from the ADC on the KALYPSO mezzanine card, data processing and data transmission via a quad optical link comprising small form-factor pluggable (SFP) transceivers. A data rate of up to 6.5 Gbps can be achieved with each channel of the quad optical link which amounts to a maximum total throughput of 26 Gbps. For the application described in this paper, only one optical link has been used for the configuration and acquisition control of the KALYPSO mezzanine card as well as raw-data streaming to the facility front-end electronics hosting the FLASH control system. The other optical links may be configured as direct low-latency communication channels to other accelerator control hardware for the implementation of fast feedbacks. The total latency for real-time data acquisition and processing of one frame with 256 pixels is less than 1 µs. A photograph of the FMC carrier equipped with the KALYPSO mezzanine card is shown in Fig. 3 ▸.

For synchronous data acquisition of the photon pulse spectra, the clock and trigger signals of the FLASH timing system are received via a twisted-pair cable with a modular connector using a low-voltage differential signalling (LVDS) standard. The clock signal is cleaned from jitter in one of the phase-locked loops (PLLs) and then provided to both the FPGA and ADC on the KALYPSO mezzanine card. The FPGA utilizes this clock and a trigger signal to control the GOTTHARD chips on the KALYPSO mezzanine card. The data acquisition can be controlled remotely and enables the recording of frames before or after the photon pulse train for background determination.

The sensitivity of the Si sensor on the KALYPSO mezzanine card depends on its bias voltage which can also be controlled remotely. The FMC carrier is equipped with a low-noise power amplifier that provides bias voltages of up to 120 V for the Si sensor via a Lemo connector. In order to reduce power losses, a DC/DC boost converter amplifies the externally supplied input voltage of 12–24 V (DC). In the case of InGaAs sensors, a second low-noise power amplifier can be used which is limited to voltages of up to 10 V. The power supply functionality is managed by a microcontroller communicating with the FPGA, and remote control of the bias voltage is realized by the connection of the FPGA to the FLASH front-end electronics via the optical link.

### 2.3. FLASH front-end electronics in MicroTCA.4 standard   

MicroTCA.4 standard (blue area in Fig. 2 ▸) is used at FLASH as front-end electronics for accelerator operation and enables electron bunch and photon pulse synchronous signal recording. The mechanics and connectivity are defined by the PICMG MicroTCA.4 specification (PICMG, 2018 ▸). A variety of fast interconnections between the electronic boards and a central processing unit (CPU) are employed on the backplane of the crate for parallel processing of larger amounts of data as well as transmission of clock and trigger signals. The front-end electronics for the communication and synchronization with the FMC carrier is based on commercially available electronic boards.

The FLASH timing system (Hidvégi *et al.*, 2014 ▸) is based on a MicroTCA.4-compliant board (NAT, NAMC-psTimer) that is used for the distribution of the accelerator clock and trigger signals to every crate via daisy chains. Inside each crate, the clock and trigger signals are distributed by the NAMC-psTimer board via dedicated timing lines of the backplane to other electronic boards. RJ-45 sockets may be used for the connection to external devices, such as the FMC carrier, via twisted-pair cables with modular connectors in LVDS standard.

The communication with the FMC carrier for the control and data transmission is realized with another MicroTCA.4-compliant board (AIES, MFMC) which comprises a FPGA (Xilinx, 7-series Artix) and two FMC slots. One of the FMC slots is equipped with a FMC board for fast SFP communication (CAENels, FMC-2SFP+) which is connected via an optical fibre to one optical link of the FMC carrier. The MFMC module comprises a 2 GB SDRAM buffer consisting of four 16-bit DDR3 chips. The 64-bit DDR bus can operate at 533 MHz resulting in a total throughput of 68.2 Gbps. PCIe lanes (4-lanes gen. 2) on the backplane of the MicroTCA.4 crate are used for data transfer to the CPU on which the data are interfaced to a server of the distributed object-oriented control system (DOOCS; Wilksen *et al.*, 2017 ▸) via direct memory access. In addition, the detector data is transferred to the shared memory of the global data acquisition system (DAQ). The DAQ collects data from electron and photon diagnostic devices together with data from user experiments. The data are sorted according to a unique pulse train number for each 10 Hz pulse train and stored for later electron bunch or photon pulse synchronous data analysis of experimental results or FEL performance evaluation.

## 3. Experimental setup   

The linear array detector KALYPSO has been installed at the variable-line-spacing (VLS) grating spectrometer (Brenner *et al.*, 2011 ▸), which is located in the photon beam distribution area of FLASH1 before three direct FEL beamlines (Tiedtke *et al.*, 2009 ▸) without monochromatization, for monitoring the spectral distributions of FEL pulses. To cover the full wavelength range of FLASH1, the VLS spectrometer is equipped with a high-energy and low-energy VLS plane grating. Both gratings are mechanically ruled on 190 mm-long silicon substrates and can be interchanged remotely. The high-energy grating with 900 grooves mm^−1^ covers a wavelength range 6–40 nm, while the low-energy grating with 300 grooves mm^−1^ covers the range 20–60 nm. In addition, the VLS spectrometer can be operated with a 510 mm-long plane mirror in case monitoring of FEL spectra is not desired. The gratings and mirror are mounted at a grazing incidence angle of 2° relative to the optics surface in order to obtain a high reflectivity at XUV and soft X-ray wavelengths and to keep the deposited energy density on the optics well below the damage threshold. The blaze angles of the gratings are optimized for zeroth-order diffraction such that the main part of the FEL radiation is transmitted to the user experiment, while approximately 1–10% of the FEL radiation intensity is dispersed into the first order for the online measurement of the spectral distributions.

The first-order diffraction is focused onto a thin Ce:YAG crystal (thickness: 0.2 mm) located in the focal plane inside the vacuum chamber of the VLS spectrometer. The FEL radiation is entirely absorbed by the Ce:YAG crystal, and the visible fluorescence light is imaged onto the radiation sensor of the linear array detector KALYPSO mounted behind a fused-silica viewport. The 1:1 imaging optics consists of two lenses (Nikon 1:1.4, *f* = 50 mm). By this, the spectral intensity distribution of the incident FEL radiation is converted into signal intensities of the 256 pixels of the radiation sensor. The wavelength range Δλ that is covered for the given sensor width of 12.8 mm varies from Δλ = 0.8 nm (for λ_central_ ≃ 5 nm) to Δλ = 2.2 nm (for λ_central_ ≃ 40 nm). This is a factor of 5 to 15 larger than the typical spectral width of about 1% of the FEL radiation. The Ce:YAG crystal together with the linear array detector KALYPSO can be moved along the focal plane for the entire wavelength range of both gratings with the help of an *x–y* translation stage in combination with a rotation stage.

## 4. Experimental results   

**Figure 4:**
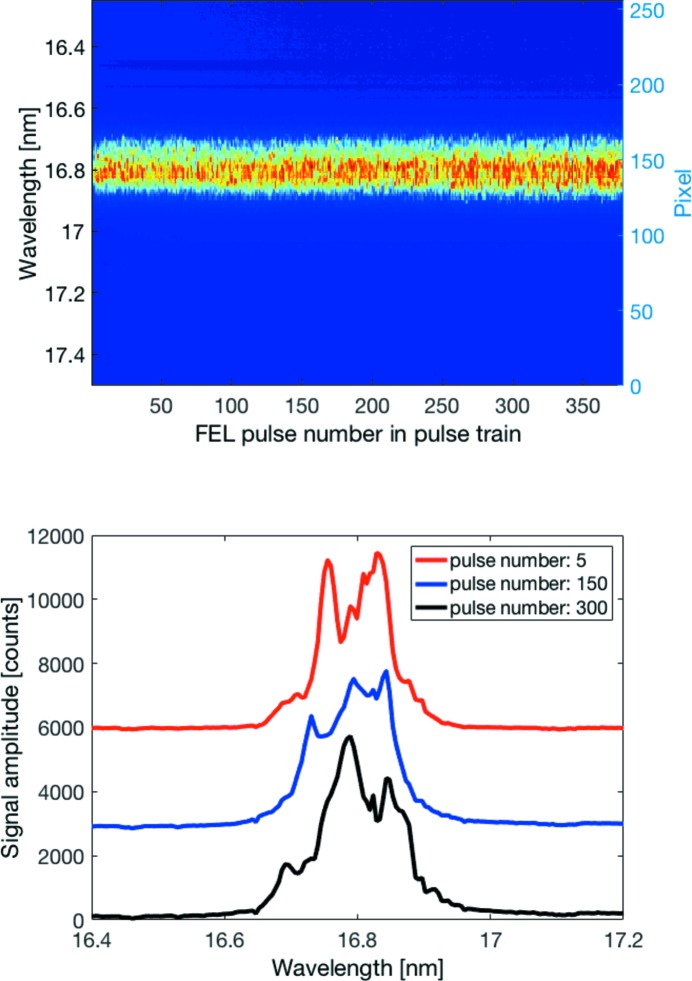
(Top) Pulse-resolved spectral distributions of a single pulse train with 380 FEL pulses recorded at a repetition rate of 1.0 MHz. The average pulse energy was 50 µJ (each pulse) and the pulse duration was about 100 fs (FWHM). (Bottom) As an example, three individual spectra are shown that have been offset vertically for better distinction. The fluctuations in the spectra result from the SASE amplification process and corroborate the need for pulse-resolved diagnostics at MHz repetition rates.

For the measurement of pulse-resolved FEL spectra for entire pulse trains, 20 frames are taken with KALYPSO before the pulse train to determine an average background frame, which is then subtracted from all frames recorded synchronously with the FEL pulses of this pulse train. The result for a single pulse train with 380 FEL pulses, recorded at the VLS spectrometer with an intra pulse-train repetition rate of 1.0 MHz, is presented as an image in the upper plot of Fig. 4 ▸. Every column of the image corresponds to a frame of the spectral distribution of a photon pulse. The rows correspond to the pixels of the KALYPSO sensor (right axis) and have been converted to wavelengths (left axis).

The pulse-resolved spectral distributions for the pulse numbers 5, 150 and 300 of the pulse train are shown in the lower plot of Fig. 4 ▸. The signal amplitudes are given in ADC counts. For better distinction, the spectra shown as blue and red curves have been offset by 3000 and 6000 counts, respectively. The number of effective bits that can be used of the 14-bit ADC amounts to 12.7 bits, *i.e.* about 6800 ADC counts.

The spectra exhibit random distributions with many spikes at a central wavelength of around 16.8 nm with a spectral bandwidth of around 1% (FWHM) which underpins the necessity of shot-to-shot detection. These spikes in the FEL spectra originate from initial charge density fluctuations in the electron bunch that lead to SASE amplification at slightly different wavelengths within a given FEL bandwidth. The number of spectral spikes is connected to the FEL pulse duration (Düsterer *et al.*, 2014 ▸), and a detailed analysis of the spectral distribution can yield information about the photon pulse duration. The large number of spikes is a fingerprint for rather long photon pulses of the order of 100 fs which has been corroborated by longitudinal electron bunch lengths measurements with a transverse deflecting structure (Behrens *et al.*, 2012 ▸). Another method to determine the FEL pulse duration is based on the precise measurement of the spike width and computation of the second-order correlation function for an ensemble of spectra (Lutman *et al.*, 2012 ▸; Engel *et al.*, 2016 ▸). However, this method can only be applied reliably for short FEL pulses with only a few spikes in the spectrum due to the given resolution of the VLS spectrometer and limited number of 256 pixels of the KALYPSO sensor.

**Figure 5:**
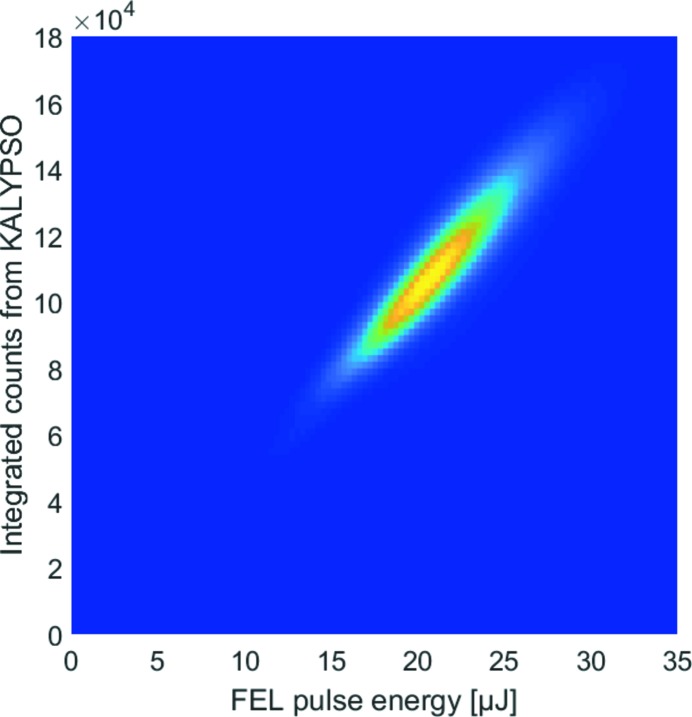
Correlation plot of more than 6 × 10^5^ integrated signal amplitudes measured with KALYPSO and the corresponding photon pulse energies measured with a GMD.

To determine the intensity response of the experimental setup including the Ce:YAG crystal and KALYPSO detector system, the sum of the signal amplitudes for all pixels of a spectrum can be compared with the absolute photon pulse energy measured with a gas-monitor detector GMD (Tiedtke *et al.*, 2008 ▸) upstream of the VLS spectrometer. Fig. 5 ▸ shows a correlation plot in which more than 6 × 10^5^ integrated signal amplitudes of individual spectra are plotted against the corresponding pulse energies recorded simultaneously with the GMD. An almost linear correlation can be identified for the range of measured values.

The central wavelengths of the individual photon pulses for the pulse train depicted in Fig. 4 ▸ are rather constant. The central wavelength (of the fundamental) is given by the undulator equation,
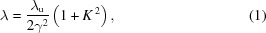
where γ = *E*_beam_/*mc* ^2^ is the relativistic factor with the electron beam energy *E*_beam_, and *K* and λ_u_ are the undulator parameter and undulator period length, respectively. The electron beam energy *E*_beam_ is the sum of all energy gains Δ*E* in the various accelerating sections, and the energy gain obtained in one section can be expressed by Δ*E* = *A*sin(φ), where *A* and φ are the amplitude and phase of the RF accelerating voltage, respectively. For each accelerating section, a fast low-level RF (LLRF) control loop regulates the amplitude and phase to operator-defined setpoints for the RF flattop region (solid green line in Fig. 1 ▸) based on RF field detection in the accelerating modules. For optimized LLRF control loop settings, as it is the case for Fig. 4 ▸, the RF amplitude variation within the flattop region is smaller than 10^−4^, and the induced variation of the central wavelength given by equation (1) is more than two orders of magnitude smaller than the typical spectral bandwidth of the FEL radiation of about 1% (FWHM).

However, especially during start-up of accelerator operation with long bunch trains, *i.e.* when the RF flattop region (see solid green line in Fig. 1 ▸) is filled with bunches, repetitive deviations from the desired central wavelength along the pulse train or instabilities of the spectral width may occur from pulse train to pulse train. The reason may be a multitude of accelerator parameters, *e.g.* bunch charge, amplitudes and phases of the RF accelerating pulses, horizontal and vertical beam positions, *etc.*, that need to be optimized and controlled along the bunch train

**Figure 6:**
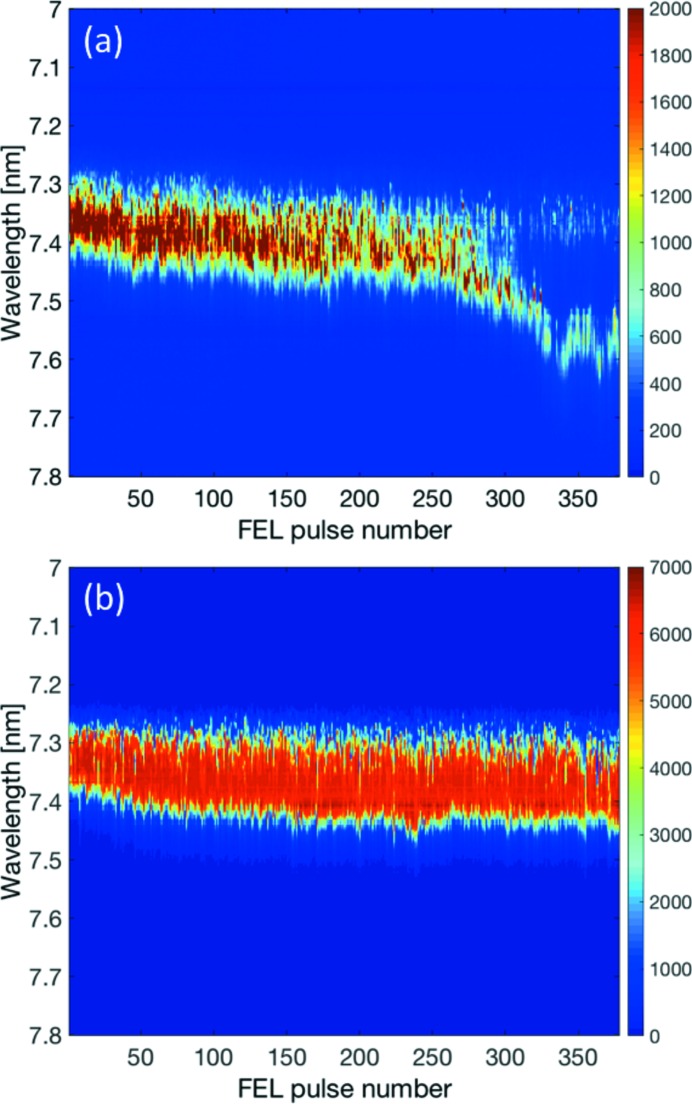
Pulse-resolved spectra of single pulse trains with 380 FEL pulses recorded at a repetition rate of 1.0 MHz. (*a*) At start-up of accelerator tuning for long bunch-train operation; (*b*) after accelerator tuning.

As an example, the image in Fig. 6(*a*) ▸ depicts the spectral distributions of a single pulse train at start-up of FEL operation for a user experiment. A slope of the central wavelength along the pulse train is visible, and strong broadening and filamentation of the spectra is apparent for pulse numbers above 250. These subtile effects occurring in only part of the pulse train strongly influence user experiments but would be difficult to detect with common detectors not capable of MHz read-out rates.

Fig. 6(*b*) ▸ shows a corresponding image after accelerator tuning and optimization of several electron beam parameters. As can be seen, the instabilities for pulse numbers larger than 250 have vanished, and the slope of the central wavelength has been reduced considerably.

In contrast to the case where a constant central wavelength over the the pulse train is desired, some user experiments (Eckert *et al.*, 2016 ▸) benefit tremendously from a larger spectral bandwidth in comparison with the single-pulse FEL bandwidth. This can be achieved by a pre-defined slope of the central wavelength along the pulse train, which can be realized with the LLRF regulation. By introducing a slope on the RF amplitude within the RF flattop region (solid green line in Fig. 1 ▸) and, therewith, a slope on the electron beam energy *E*_beam_, a shift of the central wavelength is induced along the pulse train according to equation (1).

**Figure 7:**
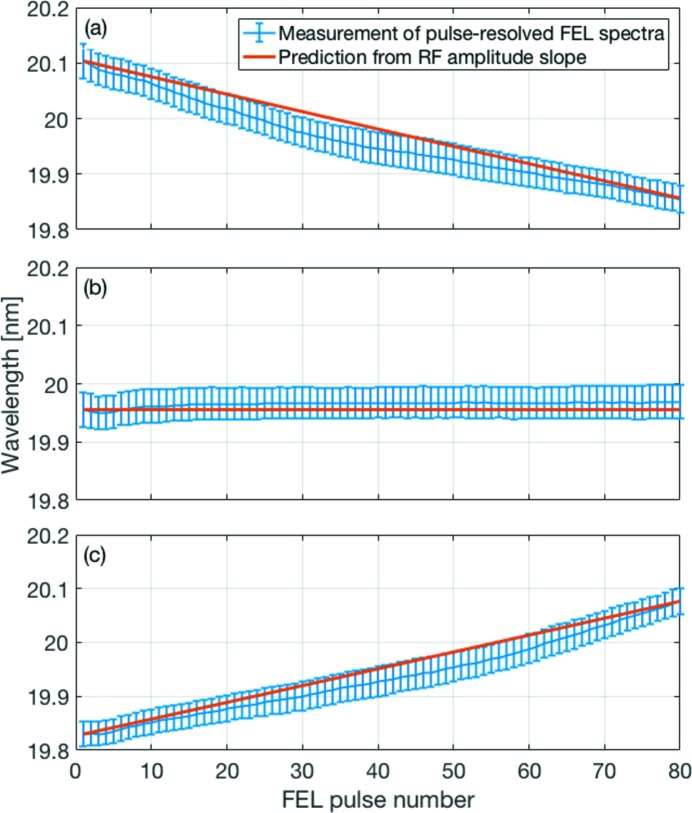
Mean value and standard deviation (FWHM) of the central wavelengths calculated for each FEL pulse number for 1000 consecutive pulse trains. (*a*) A linear slope with increasing setpoints has been applied to the RF amplitude; (*b*) constant setpoint for the RF amplitude; (*c*) a linear slope with decreasing setpoints has been applied to the RF amplitude.

The effect of an RF amplitude slope on the central wavelength is demonstrated in Fig. 7 ▸ for three different cases. In Fig. 7(*a*) ▸, a linear slope of 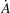 = 20 kV µs^−1^ was applied to the setpoint of the RF amplitude in the last accelerating section. This accelerating section was chosen as it has negligible impact on the formation of the longitudinal electron bunch profile which in turn would have an effect on the SASE process. The blue curve shows the mean value of the central wavelengths for each FEL pulse number averaged over 1000 pulse trains, and the error bars correspond to the standard deviation (FWHM). The central wavelength shifts to shorter wavelengths due to the increase in electron beam energy. For demonstration, the accelerator was operated with FLASH1 bunch trains of 80 bunches at a repetition rate of 500 kHz, *i.e.* a total increase in electron beam energy of 8 MeV (or relative increase of 1.4% at a mean beam energy of *E*_beam_ = 565 MeV) was introduced along the bunch train. The predicted shift in wavelength due to the slope in electron beam energy, which is depicted as a red curve for comparison, has been calculated with equation (1) and normalized to the wavelength of the first photon pulse. The measured wavelengths undershoot the predicted curve in the centre of the bunch train at a distance of about the standard deviation (FWHM) of the central wavelength. The total spectral bandwidth for the entire pulse train induced by the slope in electron beam energy is about a factor of five larger than the standard deviation (FWHM) of the central wavelength and about a factor of two larger than the typical spectral bandwidth of about 1% for a single FEL pulse.

Fig. 7(*b*) ▸ depicts the standard situation of a flattop for which the LLRF control loop regulates to constant setpoints for the RF amplitude and phase. A small dip in wavelength is noticeable for the first five FEL pulses. The origin might be a transient behaviour of the first electron bunches in the bunch train that can be caused by various effects during bunch generation and acceleration. For pulse numbers larger than five, both the mean values of the measured wavelengths and predicted curve are almost constant, and the LLRF regulation seems to work best in this case.

The case of a decreasing setpoint for the RF amplitude with a linear slope of 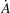 = −20 kV µs^−1^ is presented in Fig. 7(*c*) ▸. A similar behaviour of the measured wavelengths as in Fig. 7(*a*) ▸ can be seen: the measured wavelengths slightly undershoot the predicted slope (red curve) calculated from the setpoints of the RF amplitude with the help of equation (1).

**Figure 8:**
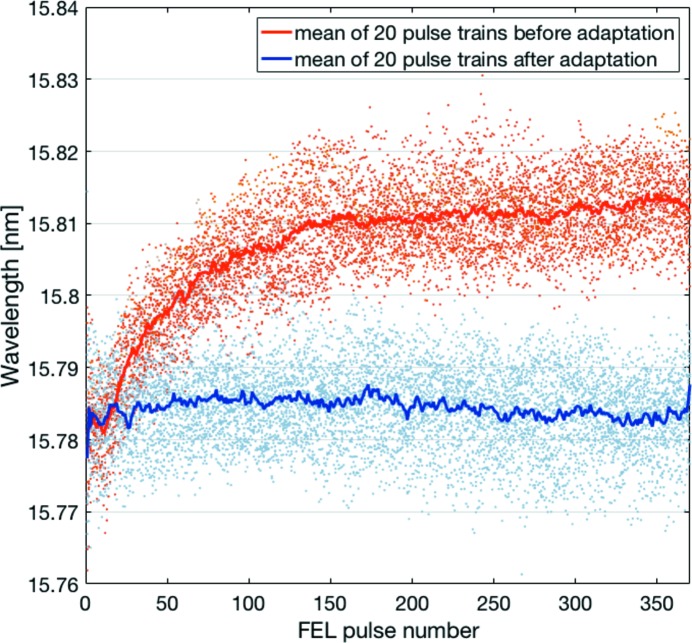
Demonstration of a photon-beam-based feedback. Mean of the central wavelengths for each FEL pulse number averaged over 20 pulse trains before (red curve) and after (blue curve) the adaptation by the LLRF regulation. The corresponding central wavelengths calculated from all individual FEL spectra are shown as light red and light blue dots.

Deviations of a measured central wavelength from a desired target wavelength can be corrected by the LLRF regulation. The differences between the measured central wavelengths and the ones predicted by the RF amplitude setpoints determine error values for each photon pulse. Assuming a linear approximation between the RF amplitude and central wavelength for small differences, these error values can be inverted and applied as an RF amplitude correction to the LLRF regulation. Since this is a pulse-train to pulse-train adaptation, only repetitive errors can be reduced. The result of such a photon-beam-based feedback for the case of a constant target wavelength along the pulse train is presented in Fig. 8 ▸. The central wavelengths calculated from the pulse-resolved FEL spectra of 20 subsequent pulse trains with 380 FEL pulses at an intra pulse-train repetition rate of 1.0 MHz are depicted as light red dots. The jitter of these central wavelengths is a result of the random spikes in the spectral distributions which originate from the SASE process (as shown in the lower plot of Fig. 4 ▸). The mean of the central wavelengths for each pulse number of the 20 pulse trains is displayed as a red curve, for which a slope along the pulse train towards longer wavelengths can be identified as a repetitive error. In order to suppress the jitter stemming from the individual FEL spectra, the mean of the central wavelengths averaged over several pulse trains has been used for an adaptive pulse-train to pulse-train feedback. The result of the feedback adaptation is shown as a blue curve, while the central wavelengths calculated for the individual FEL spectra of 20 subsequent pulse trains are depicted as light blue dots. As can be seen, the slope of the mean central wavelengths along the pulse train has been adapted by the feedback to the constant target wavelength. In general, any wavelength distribution along the pulse train can be applied in the adaptive feedback within certain bandwidths and power limitations of the superconducting accelerator.

## 5. Summary   

At the FLASH facility, the linear array detector KALYPSO has been installed at the VLS spectrometer as online photon diagnostics for spectral distributions of FEL radiation pulses. The KALYPSO detector has been synchronized to the FLASH timing system, and pulse-resolved spectra have been recorded at a frame rate of 1.0 MHz for pulse trains with up to 380 FEL pulses. The current version of KALYPSO can be equipped with Si or InGaAs microstrip sensors with 256 pixels for the detection of visible or near-infrared radiation. To be compatible with the FLASH front-end electronics in MicroTCA.4 standard, a FPGA-based carrier board has been developed for data acquisition and transmission. Fast data processing in the FPGA can be utilized for data reduction or fast feedback applications with a latency below 1 µs. The MHz read-out rate with low latency makes the linear array detector KALYPSO an excellent candidate for applications of one-dimensional profile measurements at high-repetition-rate FELs or synchrotron radiation facilities.

The random spectral distributions with many spikes, which are inherent in the SASE FEL radiation and discernible in the individual pulse-resolved spectra recorded with KALYPSO, underpin the need for shot-to-shot detection with synchronous read-out at MHz frame rates. For later post analysis and interpretation or sorting of data taken in user experiments, the pulse-resolved spectra are saved to a central data acquisition system synchronously with data from user experiments. In addition, by real-time monitoring of pulse-resolved FEL spectra of entire pulse trains, KALYPSO has also become a valuable tool for accelerator tuning of long pulse-trains at FLASH1.

By introducing a slope on the RF amplitude within the RF accelerating pulse, a shift of the central wavelength over the pulse train can be realized. As a result, the total spectral bandwidth over the pulse train could be increased to about 2% compared with the typical spectral bandwidth of about 1% for single FEL pulses. It has been demonstrated that the LLRF regulation can also be used to adapt the measured central wavelengths along a pulse train to a chosen target wavelength in case of repetitive slope errors. Such a photon-beam based adaptive feedback has been applied at FLASH for the first time.
